# Pro-enkephalin in plasma of surgical icu-patients with sepsis - a pilot study

**DOI:** 10.1186/2197-425X-3-S1-A256

**Published:** 2015-10-01

**Authors:** S Doemming, T-P Simon, A Humbs, L Martin, C Bruells, O Hartmann, J Struck, A Bergmann, G Marx, T Schuerholz

**Affiliations:** Department of Intensive Care and Intermediate Care, University Hospital RWTH Aachen, Aachen, Germany; Sphingotec GmbH, Hennigsdorf, Germany

## Introduction

Enkephalins are opioid peptides and enkephalin and its precursor pro-enkephalin (pro-ENK) are found to be widely distributed in organ tissues including the kidney [1]. Pro-ENK is a predictor for acute kidney injury (AKI) in patients after cardiac surgery and can detect severity of AKI in patients admitted to the emergency department with sepsis [2, 3]. In surgical ICU-patients with sepsis, information on the suitability of pro-ENK to determine kidney function is lacking.

## Objectives

To evaluate kidney function by measuring pro-enkephalin in surgical ICU-patients with sepsis, severe sepsis or septic shock.

## Methods

In a prospective, observational trial, we included 42 consecutive ICU patients after major surgery with clinical signs of sepsis according to ACCP/SCCM definitions. Plasma samples to determine pro-ENK were drawn within 16 hours after diagnosis of sepsis. Laboratory and clinical parameters were recorded. Values are expressed as median and interquartile ranges (IQR), or counts and percentages as appropriate. Group comparisons of continuous variables were performed using Kruskal-Wallis test. Biomarker data were log-transformed. Spearman rank-order correlation was applied to continuous variables. A p < 0.05 was considered significant.

## Results

Patients (67% male) were 73 (IQR 58-77) years old and had a body mass index of 26.0 (IQR 23.4-29.3) kg/m². Lengths of stay (LOS) in ICU was 6 (IQR 2-18) days and in hospital 18 (IQR 11-26) days. Of 42 consecutive ICU patients, eight patients had sepsis, 19 severe sepsis and 15 suffered from septic shock. Pro-ENK is increased, but does not differ significantly according to disease severity (table 1, p = 0.069).

**Table 1:** *Pro-ENK* concentrations.

Subgroup	n	Median pmol/L)	IQR(pmol/L)
Sepsis	8	44.3	40.8 - 57.6
Severe sepsis	19	85.6	37.1 - 163.6
Septic shock	15	86.0	58.7 - 146.8

Pro-ENK concentrations in sepsis, severe sepsis and septic shock patients (p = 0.069).

**Figure 1:**
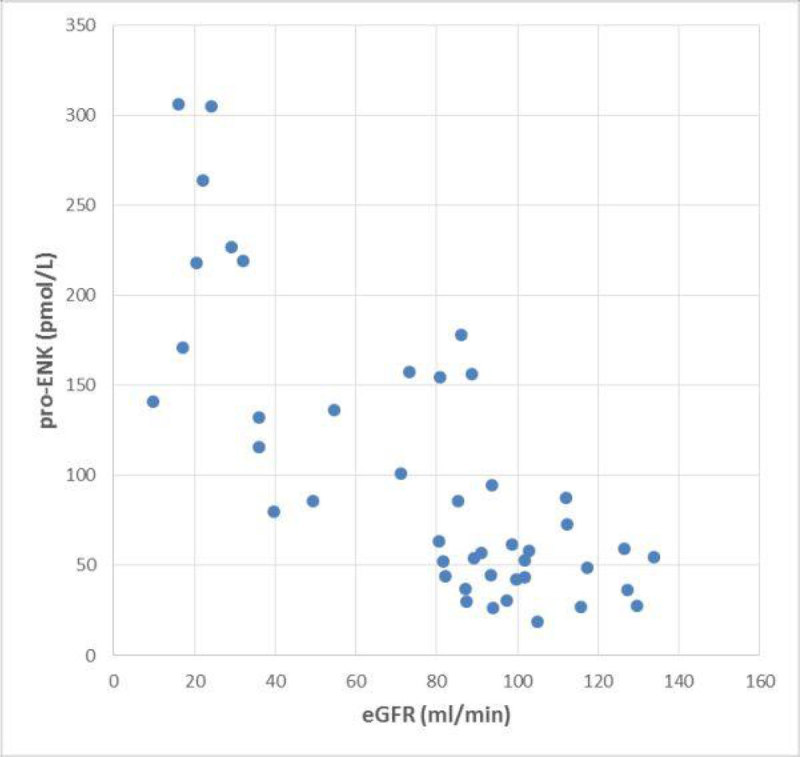
**Correlation between pro-ENK and eGFR.**

In contrast, pro-ENK showed a negative correlation with eGFR (r = -0.74, Figure 1) and a positive correlation with serum creatinine (r = 0.51).

Spearman rank correlation coefficient r = -0.74 (95% CI -0.84 to -0.57, p < 0.0001).

**Figure 2:**
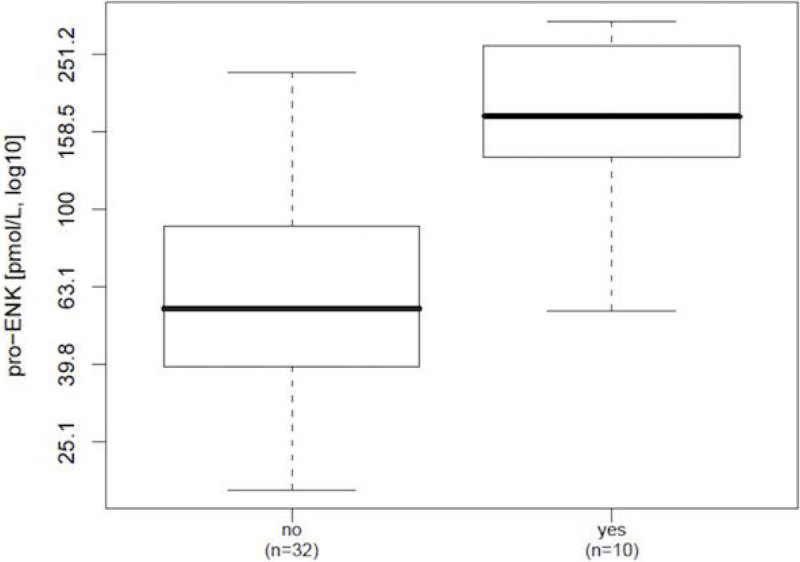
**Renal dysfunction.**

**Figure 3:**
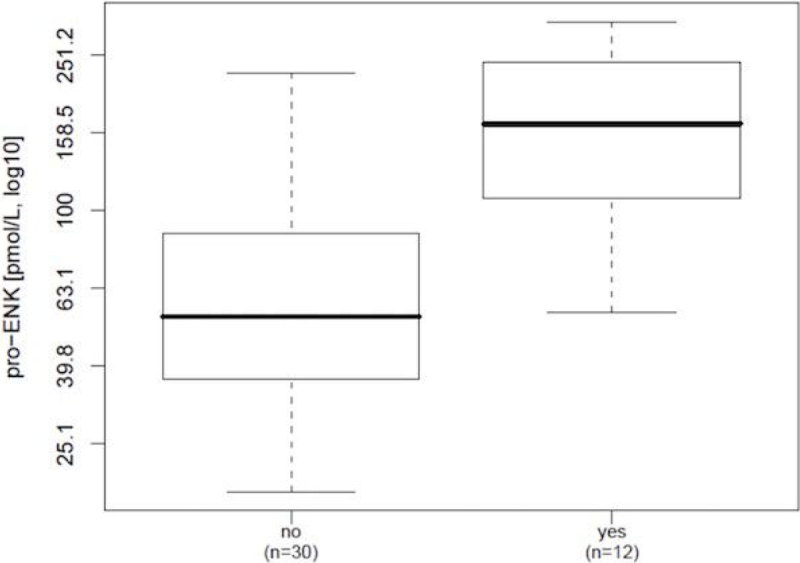
**Metabolic acidosis.**

Higher pro-ENK levels were found in patients with renal dysfunction and metabolic acidosis (p = 0.0002 and p = 0.003, respectively; Figure 2 and 3).

Linear regression including age, gender, BMI, eGFR, renal dysfunction and metabolic acidosis revealed a strong influence of eGFR on pro-ENK plasma levels (partial R = 0.35, p = 0.0002) and some influence of metabolic acidosis (partial R = 0.19, p = 0.029), while the other factors did not gain significance.

## Conclusions

Pro-enkephalin is increased in surgical ICU-patients with suspected sepsis and renal dysfunction and correlates significantly with the eGFR. Thus, pro-enkephalin may serve as laboratory parameter to monitor kidney function in surgical patients with suspected sepsis.

## Grant Acknowledgment

This study was supported by a restricted grant of Sphingotec, Hennigsdorf, Germany
